# Empathy and psychopathology in children and adolescents: the role of parental mental illness and emotion regulation

**DOI:** 10.3389/fpsyt.2024.1366366

**Published:** 2024-04-08

**Authors:** Arleta A. Luczejko, Klara Hagelweide, Rudolf Stark, Sarah Weigelt, Hanna Christiansen, Meinhard Kieser, Kathleen Otto, Corinna Reck, Ricarda Steinmayr, Linda Wirthwein, Anna-Lena Zietlow, Christina Schwenck, the COMPARE-family research group, Stracke

**Affiliations:** ^1^ Department of Clinical Child and Adolescent Psychology, Justus Liebig University Giessen, Giessen, Germany; ^2^ Department of Rehabilitation Sciences, Technical University Dortmund, Dortmund, Germany; ^3^ Department of Psychotherapy and Systems Neuroscience, Justus-Liebig University Giessen, Giessen, Germany; ^4^ Department of Psychology, Clinical Child and Adolescent Psychology, Philipps University Marburg, Marburg, Germany; ^5^ Institute of Medical Biometry, University of Heidelberg, Heidelberg, Germany; ^6^ Department of Work and Organizational Psychology, Philipps-University Marburg, Marburg, Germany; ^7^ Department of Psychology, Ludwig-Maximilians-Universität München, Munich, Germany; ^8^ Department of Psychology, Technical University Dortmund, Dortmund, Germany; ^9^ Clinical Child and Adolescent Psychology, Department of Psychology, Technische Universität Dresden, Dresden, Germany

**Keywords:** transgenerational transmission of mental disorders, parents with mental illness, children of parents with mental illness, empathy, emotion regulation

## Abstract

**Objective:**

Although empathy is known to be a strength, recent studies suggest that empathy can be a risk factor for psychopathology under certain conditions in children. This study examines parental mental illness as such a condition. Further, it aims to investigate whether maladaptive emotion regulation (ER) mediates the relationship between empathy and psychopathological symptoms of children.

**Methods:**

Participants were 100 children of parents with a mental illness (55% female) and 87 children of parents without a mental illness (50% female) aged 6 - 16 years and their parents.

**Results:**

Greater cognitive empathy was related to more psychopathological symptoms in COPMI, but not in COPWMI. In addition, in COPMI maladaptive ER mediated this relationship. In contrast, greater affective empathy was associated with more psychopathological symptoms regardless of whether parents had a mental illness.

**Conclusion:**

Our findings highlight the importance of implementing preventive programs for COPMI that specifically target the reduction of maladaptive ER.

## 1 Introduction

### 1.1 Children of parents with a mental illness

It is estimated that around 25 percent of children live in a household with at least one mentally ill parent (1–4). Children of parents with a mental illness (COPMI) are considered a high-risk population for the development of psychological disorders. Compared to children of parents without mental illness (COPWMI), COPMI have not only an increased lifetime risk to develop a mental illness themselves (5–7), but they are also at risk for multiple psychological and developmental disadvantages. In particular, COPMI have more subclinical internalizing and externalizing symptoms (8, 9). Thus, a transgenerational transmission of mental disorders (TTMD) can be assumed. That makes COPMI a target group for selective prevention programs.

In the TTMD model (5), different transmission factors and mechanisms and their interplay are assumed to underlie the transmission of mental disorders. Parent- and child-related factors display a promising target for preventive measures. However, the impact of the single factors is not sufficiently tested yet. In this context, empathy is of special interest, as besides the positive role it displays in many fields of interpersonal interaction, it has been shown to be a risk factor for psychopathology under certain conditions (10).

### 1.2 Empathy

Empathy broadly refers to reactions of an individual to another person’s experiences (11). Research has shown that empathy is a multidimensional construct (11), consisting of both cognitive and affective facets (12). Cognitive empathy has been conceptualized as the ability to take over another person’s perspective and require an understanding of affect related motives, thoughts and feelings of a person [Birnie et al., (13)]. In contrast, affective empathy includes the ability to connect with the emotional state of another person (13) and sharing of affective states and feeling of concern for others (11, 14). A special subset of empathy is sympathy (15). Baron-Cohen and Wheelwright (15) define sympathy as feeling an emotion after seeing/learning another person’s distress which in turn moves one to alleviate the suffering of the other. It can involve elements of both cognitive and affective empathy (15). In contrast to sympathy, personal distress is a self-focused aversive affective reaction accompanied by the motivation to reduce the own distress (16).

### 1.3 Empathy and psychopathology

Typically, empathy is seen as a strength and various studies reveal relations between empathy and positive outcomes in various functional areas in children. For example, responding empathically to others is associated with adaptive functioning and social competence (17, 18), popularity among peers (19, 20), and is linked to children’s academic success (21). In addition, previous studies have shown a negative relationship between empathy and aggression or externalizing problems (22, 23).

However, recent theoretical and empirical literature suggests that empathy might also be associated with certain risks (10, 24, 25). Particularly, affective empathy is positively associated with internalizing symptoms. This association seems to apply not only to children and adolescents in clinical (26, 27) but also to non-clinical (28–30) samples. In contrast, studies investigating cognitive empathy are less consistent. Whereas studies with healthy children (29) indicated that low cognitive empathy is associated with more psychopathology, studies investigating clinical samples of children did not find evidence for a relationship (26, 27). Empirical data on empathy and its effect on psychopathology in COPMI is limited. Studies investigating whether higher psychopathological symptoms in COPMI (8, 9, 31) are associated with higher or lower empathy levels are inconsistent. On the one hand, children of depressed mothers had both higher prevalence of psychopathology and lower affective empathy levels than children of mothers without depression (32). However, it must be noted that in this study more than half of the children had a mental illness themselves. In contrast, in the study conducted by Tully and Donohue (24) children of chronically depressed mothers and healthy mothers did not differ significantly in cognitive and affective empathy. Interestingly, higher levels of empathy (cognitive and affective) were related to greater internalizing problems in children of chronically depressed mothers (depressed for 36 months) only (24). At the same time, affective and cognitive empathy and internalizing symptoms were unrelated in children of mothers with shorter (12 or 24 months) depression and in children of mothers without depressions.

Zahn-Waxler and Van Hulle (33) suggested distinct pathways through which empathy can be adaptive or maladaptive in children: Unfavorable conditions in the early family environment contribute to a maladaptive pathway. For example, in case of parental depression, empathy can lead to anxiety, sadness and guilt because the child develops self-blame cognitions followed by pathogenic guilt. Pathogenic guilt in children, in turn, heightens the risk for developing depression (33). In this line, Tone and Tully (10) proposed that different moderators affect the development and impact of affective and cognitive empathy. These moderators in conjoint with emotion regulation difficulties lead to personal distress and guilt, resulting in an increased risk for internalizing problems. Parental mental depression is considered to be such a moderator. Thus, the tendency being cognitively empathic could be accompanied by attempts to understand the mother’s emotions and their fluctuations (24). Subsequent inaccurate assumptions of responsibility for the sadness of the parents, in turn, increases the risk of guilt and self-blame if the child is not able the regulate it (24). In regard to affective empathy, Tully and Donohue (24) suggested that affective empathic sensitivity in COPMI can lead to internalizing symptoms via deficits in emotion regulation (ER) strategies and personal distress, as well. Children with high affective empathy tendencies may have unregulated arousal, hypervigilance and distress in response to the depression of the mother if they cannot regulate their emotions effectively. The authors further argue that affective and cognitive empathy, while related, seem to function independently. Thus, affective empathy does not predict cognitive empathy or vice versa (24). While the models and assumptions described above are limited to parental depression only, it could apply to other parental mental disorders as well, as COPMI suffer from self-blame, misplaced responsibility or other dysfunctional cognitions regardless of the parent’s specific diagnosis (34, 35).

Inconsistent findings regarding the association between empathy and psychopathology could be explained, inter alia, by mediating factors such as ER. Thus, empathy and psychopathological symptoms may not be directly related, but rather indirectly through ER. ER comprises processes that influence the incidence, kind, intensity, and duration of emotions as well as their effects on feelings and behaviors (36, 37). Strategies of ER can be adaptive (e.g. cognitive re-appraisal, problem solving, acceptance, distraction) if they increase positive or decrease negative emotions, or be maladaptive (e.g. rumination, suppression), having the opposed effect (38).

In addition to empathy, ER has been shown to be associated with psychopathology in children (39–42). In particular, the increased use of maladaptive ER strategies is associated with psychopathology in mental disorders of many kinds (39, 43). Studies investigating ER in COPMI revealed deficits in ER in COPMI versus COPMWI (8, 44–46).

It has been theoretically proposed that ER play a crucial role in the impact of empathy (47). By perceiving another individual’s state, an emotion state in the observer is generated. The latter is a function of the observer’s level of cognitive and affective empathy and is subject to the emotion regulatory process of the observer (48). The assumption is, that deficits in ER lead to higher levels of personal distress and lower levels of sympathy when confronted with another individual’s negative emotional state. Empirical studies further indicate that cognitive and affective empathy may be differentially related to ER. In adult community samples it has been shown that maladaptive ER is negatively related to cognitive empathy (25, 49, 50) and positively related to affective empathy (25, 49, 51). Studies investigating empathy and ER in healthy children are lacking to date. However, the results with adult community samples are expanded through single studies on either ER or empathy investigating clinical samples. Children with autism spectrum disorder show impairments in cognitive empathy (52), and also an increased use of maladaptive regulation strategies (53). Adult patients with borderline personality disorder are also characterized by having difficulties in ER (54), but accompanied by increased affective empathy (55).

In summary theoretical and empirical literature mainly support the association between high affective and low cognitive empathy and maladaptive ER on the one hand and high affective and low cognitive empathy and psychopathology on the other hand. However, the mediating effect of maladaptive ER on this relationship is barely examined. To the best of our knowledge, there is only one study that has investigated this relationship in an adult community sample (25). MacDonald and Price (25) showed that maladaptive ER mediated the relationship between affective empathy and internalizing symptoms. However, cognitive empathy and internalizing symptoms were not related.

It can be stated that there is a lack of studies investigating the role of parental mental illness as risk factor for the maladaptive pathway from empathy to psychopathological symptoms in children. In addition, the pathway by which empathy might contribute to psychopathological symptoms in COPMI has not yet been empirically studied. It is important to note, that all studies and models in this research area refer to internalizing symptoms. However, COPMI have an increased risk to develop not only internalizing but also externalizing symptoms (8, 9, 31). Further, the use of maladaptive ER strategies in children and adolescents is associated with both internalizing and externalizing symptoms (56). Therefore, the tendency being highly empathic could also be associated with externalizing symptoms in COPMI. Identifying the mechanisms of risk is of clinical importance since the reduction of maladaptive ER strategies or self-blaming thoughts and guilt could be targeted in preventive interventions and buffer the impact of parental mental illness on children.

### 1.4 The current study

**Figure 1:**
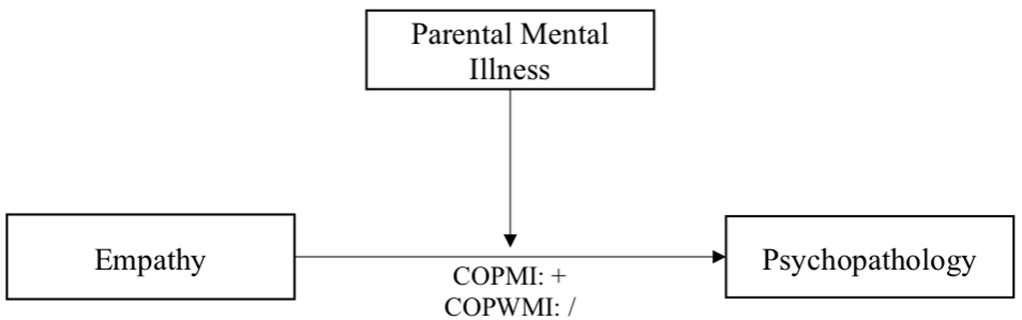
Diagram of proposed moderation model of the relationship between empathy and psychopathology.

In line with the theoretical background and it´s gaps, the first aim of the current study is to examine the moderating role of parental mental illness on the relationship between both cognitive and affective empathy and psychopathological symptoms in children. According to the results of the only existing study on this topic of Tully and Donohue (24), we hypothesize that empathy (cognitive & affective) is positively related to internalizing symptoms in COPMI and unrelated in COPWMI. We assume the same pattern for externalizing symptoms (see **Figure 1** for demonstration).

**Figure 2:**
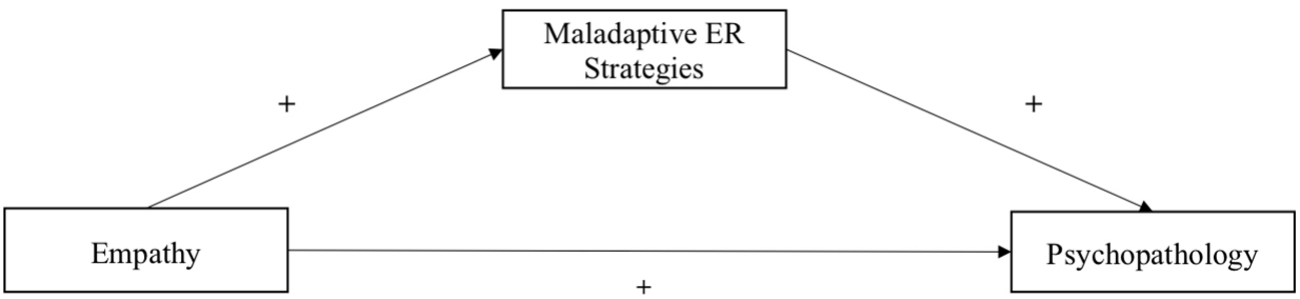
Diagram of proposed mediation model of the relationship between empathy and psychopathology.

The second aim is to investigate the mediating effect of maladaptive ER strategies on the relationship between cognitive/affective empathy and psychopathological symptoms in children. We examined maladaptive ER strategies because psychopathology is rather associated with maladaptive ER (39). The use of maladaptive ER strategies in turn is positively associated with personal distress (57, 58). Both are suggested to underlie the maladaptive pathway of empathy (10). If the moderating effect of parental mental illness is confirmed, we will test the mediations separately for COPMI and COPWMI. We assume, that maladaptive ER mediate the relationships in COPMI only. We hypothesize that higher affective/cognitive empathy is related to more maladaptive ER strategies and more maladaptive ER strategies are associated with more psychopathological symptoms in this specific group. We will test all hypotheses separately for cognitive and affective empathy (see **Figure 2** for demonstration).

## 2 Method

The present study is part of the project Children of Mentally Ill Parents At Risk Evaluation and its add-on project COMPARE-Emotion. The projects are described in detail in the study protocols (1, 59). All procedures performed were in accordance with the ethical standards of the institutional research committee and with the 1964 Helsinki declaration and its later amendments or comparable ethical standards. The study was approved by Local Ethics Committees of participating Universities. Informed consent was obtained from all individual participants included in the study.

### 2.1 Participants

Two hundred parents with mental illness signed informed consent for participating in the add-on project COMPARE-Emotion. However, complete data sets were only available from 122 independent parent-child dyads. Of these dyads, in turn, other information from the COMPARE-Family project such as psychopathology of parents or children were missing (*N*=22). Eighty-seven parents without mental illness signed informed consent for COMPARE-Emotion. In the end, for the current study complete data sets of n = 187 independent parent-child dyads including 100 COPMI and 87 COPWMI were available. Children ranged from six to sixteen years (*M* = 10.42, *SD* = 2.53) in age and included 82 males (44%). COPMI and COPWMI groups did not differ in child age, child gender and parents’ age. Children’s age was evenly distributed across males and females, *t*(185) = 1.31, *p* = .190, Cohen’s *d* = .19. Furthermore, the socioeconomic status (SES) of COPMI (*M* = 4.69, *SD* = .99) was lower than of COPWMI (*M* = 6.06, *SD* = .85). For demographic characteristics of participants separately for COPMI and COPWMI see **Table 1**. As noted in **Table 2**, 45% of mentally ill parents had a Depressive Disorder as primary diagnosis. The number of comorbid diagnoses in parents with mental illness ranged between 0 - 5 (*M* = 1.12, *SD* = 1.19), and average severity of the primary diagnosis was six (*SD* = 1.03, range from 3 - 8).

**Table 1:** Demographic characteristics of participants and means and standard deviations of psychopathological symptoms of children and parents, empathy, and maladaptive ER strategies of children.

	COPMI (*N* = 100)	COPWMI (*N* = 87)	*t*(185)/*χ* ^2^(1)	*p*	Cohen’s *d/φ*
Children					
Age	10.20 (2.56)	10.66 (2.45)	1.24	.218	.18
Gender (female, %)	55 (55.00)	50 (57.47)	.12	.734	-.02
CBCL ext (T-score)	52.11 (9.35)	47.94 (7.64)	-3.31	.001	-.79
CBCL int (T-score)	56.01 (9.95)	49.08 (7.22)	-5.38	<.0001	-.49
Cognitive Empathy	20.47 (4.71)	15.08 (4.18)	-8.22	<.0001	-1.21
Affective Empathy	21.06 (3.56)	15.14 (3.99)	-10.71	<.0001	-1.57
Maladaptive ER	25.81 (6.52)	17.87 (6.97)	-8.04	<.0001	-1.17
Parents					
Age	42.02 (6.31)	43.33 (5.88)	1.42	.158	.21
Gender (female, %)	76 (76.00)	72 (82.75)	1.29	.257	-.08
SES	4.65 (.99)	5.99 (.87)	9.76	<.001	1.43
BSI GSI (T-score)	60.81 (9.36)	43.41 (7.86)	-13.65	<.0001	-2.00

CBCL, Child Behavior Checklist (inter- and externalizing symptoms); ER, Emotion regulation; SES, Socioeconomic status; BSI, Brief Symptom Inventory; GSI, Global Severity Index.

**Table 2:** Classifications of current primary diagnoses in parents with mental illness.

	*N*	%
Schizophrenia Spectrum an Other Psychotic Disorders	2	2.0
Bipolar and Related Disorders	1	1.0
Depressive Disorders	45	45.0
Anxiety Disorders	19	19.0
Obsessive-Compulsive and Related Disorders	1	1.0
Trauma- and Stressor-Related Disorders	20	20.0
Somatic Symptom and Related Disorders	7	7.0
Feeding and Eating Disorders	3	3.0
Sleep-Wake Disorders	1	1.0
Personality Disorders	1	1.0

### 2.2 Participant recruitment and study inclusion criteria

COPMI were recruited as part of a randomized controlled multicenter RCT-study for COPMI in Germany (COMPARE-Family) (1, 59). Patients were primarily recruited from the University outpatient clinics at each study site. In the study center in Giessen patients were recruited in addition by mailings of randomly picked addresses of families with children in the corresponding age range provided by the local registry office, public advertisement (flyer, newspaper), inpatient psychiatric clinics (COPMI) and the University’s internal mailing list. COPWMI were recruited as part of the add-on project COMPARE-Emotion in addition via the research group’s database of former study participants. Inclusion criteria for COMPI were: (a) between 6-16 years of age, (b) parent with a mental illness according to the Diagnostic and Statistical Manual of Mental Disorders (DSM-5) (60). For COPWMI inclusion criteria were (c) parents without mental disorders and without psychotherapeutic treatment in the last 5 years or after the child was born. Exclusion criteria were (a) insufficient German language skills of children and the parents, (b) severe impairment of the children requiring comprehensive treatment, (c) parental outpatient or inpatient treatment while participating in the study, or continuous use of benzodiazepines.

The study was approved by the local ethics committee. All participants and their parents gave written informed consent. While the families of the COPWMI group only took part in the add-on project once, the assessment was repeated for the families of the COPMI group at three measurement points (59). From the COPMI group the data of the first assessment point of the study were analyzed.

### 2.3 Measures

#### 2.3.1 Socioeconomics tatus

To assess the SES of COPMI and COPWMI, professional status and net household income were translated into numbers between 1 and 7 according to the scales used in the second wave of the KiGGS study (61). The mean of both values was computed.

#### 2.3.2 Brief symptom inventory

The mental impairment level in parents of COPMI and COPWMI was assessed using the Global Severity Index (GSI) of the BSI. The BSI is a self-report questionnaire and contains 53 items that are rated on a 5-point Likert scale (0 = “not at all” to 4 = “very much”). Internal consistency is very good for the GSI (Cronbach’s alpha = 0.97) (62).

#### 2.3.3 Interpersonal reactivity index

The IRI (11, 63) is a self-report questionnaire containing 28 items on four 7-item subscales. Each subscale addresses a separate aspect of the global trait of empathy using a 5-point Likert scale (1 = “does not describe me well” to 5 = “describes me very well”). While the mean of the subscales “fantasy” and “perspective taking” display the cognitive empathy, the mean of the subscales “empathic concern” and “personal distress” capture the affective empathy. For all scales, satisfactory test-retest reliabilities ranging from.61 to.81 as well as internal reliabilities ranging from.70 to.78 have been reported (63). Cronbach’s α of observed mean scores showed acceptable values, ranging from.67 to.77.

##### 2.3.3.1 Outcome measures

###### 2.3.3.1.1 Child behavior checklist

We applied the German version of the parent-report measure CBCL 6-18R (64) from the Achenbach system of empirically based assessment in COMPI and COPWMI. It consists of 99 items assessing problems of children between the age of 4 and 18 years using a 3-point Likert scale (0 = “do not agree” to 2 = “agree”). The items constitute three superordinate scales “external, internal and total problems”, which constitute as dependent variable. Internal consistency of the superordinate scales is reported as good to excellent (Cronbach’s alpha = .85 -.93) (64).

*Questionnaire to Assess Emotion Regulation in Children and Adolescents (FEEL-KJ).* The FEEL-KJ by Grob and Smolenski (2005) assesses ER strategies concerning fear, sadness, and anger among children and adolescents using a 5-point Likert scale (1=“almost never” to 5 = “almost always”). While the original version consists of 90 items, we applied the self-report short version of the FEEL-KJ (65) in COMPI and COPWMI. It consists of 30 items in total, 14 items of which measure adaptive and 10 items maladaptive strategies. Each item of the short version integrates the three emotions of the original version into a superordinate emotional state (e.g., “If I am unhappy (sad, angry, anxious), I do not want to see anybody”). The scale of maladaptive strategies was used. No reliabilities are reported for the short version of the self-report, yet the internal consistency for the original version of the self-report is good for the higher-order scale maladaptive (Cronbach’s alpha = .82) ER strategies with two-week test-retest-reliabilities *r*_tt_ = .88 for maladaptive ER strategies (66).

##### 2.3.3.2 Eligibility measures

###### 2.3.3.2.1 Diagnostic interview for mental disorders

The DIPS (67) was used to assess whether parents of COPMI met the diagnostic criteria for study inclusion. The DIPS is a semi-structured diagnostic interview to determine mental disorders according to the DSM-5 (60, 67). Parents of the COPWMI were only interviewed if the BSI was above the cut-off value (T_GSI_ ≥ 62). Previous studies report high inter-rater reliability using the instrument (.72 < κ < 0.92) and test-retest reliabilities mostly in the range of.62 to.94 (68).

###### 2.3.3.2.2 Diagnostic interview for mental disorders during childhood and adolescence

The diagnostic assessment of the children was conducted using the parent report of the Kinder-DIPS (69). The Kinder-DIPS is a structured diagnostic interview to determine mental disorders from age six to adulthood according to DSM-5 (70). report good to very good interrater reliabilities for the self- and parent-report of the Kinder-DIPS. Diagnostic interviews for COPMI were done by default. In the COPWMI group, parents were only interviewed if the value of total problems of the CBCL was above the cut-off value (T_CBCLSum_ ≥ 60).

### 2.4 Analysis strategy

All statistical analyses were performed using SPSS version 27 (71). For the moderation and mediation analyses, the PROCESS tool was used (72). The analytical strategy included preliminary analyses of possible differences between groups (COPMI vs. COPWMI) in the study variables according to demographic characteristics to address the need for potential confounding variables in the subsequent analyses. For both moderation and mediation analyses unstandardized path coefficients are reported.

Aim 1: Moderation analyses were run to determine whether the relationship between child empathy and child psychopathological symptoms is moderated by parental mental illness [model 1 of the PROCESS Tool, Hayes (72)]. Separate moderation analyses for affective/cognitive empathy and internalizing/externalizing symptoms were calculated. The relationships of all variables involved in the moderation analyses were approximately linear, as assessed by visual inspection of the scatterplots after LOESS smoothing. Further, observations were independent. Since we used a robust method for the analyses, we dispense with checking normal distribution and heteroscedasticity (72).

Aim 2: Whether child maladaptive ER strategies mediate the relationship between child empathy and child psychopathological symptoms was analyzed by moderation analyses [model 4 of the PROCESS Tool, Hayes (72)]. Bivariate correlations were calculated to determine the relations between the study variables. We calculated mediation analyses with maladaptive ER strategies as mediator separately for internalizing and externalizing symptoms. The relationships of all variables involved in the mediation analysis were approximately linear, as assessed by visual inspection of the scatterplots after LOESS smoothing. Further, observations were independent. Since we used a robust method for the analyses, we dispense with checking normal distribution and heteroscedasticity (72). Indirect effects were estimated using the bootstrapping technique with 5000 bootstrap samples and 95% BC confidence intervals. The mediation model was determined to be significant if the 95% BC confidence interval did not contain zero. Since the groups differed in SES (*p* <.001), this variable was included as a covariate in each analysis conducted with the total sample.

## 3 Results

Descriptive data for the independent and dependent variables are shown in **Table 1**. The results show that COPMI have more psychopathological symptoms than COPMWI, both internalizing and externalizing symptoms. COPMI also showed higher scores on maladaptive ER strategies and both cognitive and affective empathy.

### 3.1 Empathy and psychopathology of children: the moderating effect of parental mental illness (aim 1)

For affective empathy as independent variable and internalizing symptoms as outcome variable, the overall model was significant, *F*(4, 182) = 8.95, *p* <.0001, predicting 16.43% of the variance. However, results did not show that parental mental illness moderates the effect between affective empathy and internalizing symptoms significantly. Following recommendations by Hayes (72), the interaction term and moderator was dropped from the model, resulting in a new linear regression model with the independent variable affective empathy. This new model revealed a significant relationship between affective empathy, *B* = .366, *p* <.001, for internalizing symptoms indicating that affective empathy predicts internalizing symptoms positively. For affective empathy as independent variable and externalizing symptoms as outcome variable, the overall model was significant, *F*(4, 182) = 3.38, *p* = .011, predicting 6.91% of the variance. However, parental mental illness did not moderate the effect between affective empathy and externalizing symptoms significantly. The followed linear regression model (see above) revealed a significant relationship between affective empathy, *B* = .217, *p* = .006, for externalizing symptoms indicating that affective empathy predicts externalizing symptoms positively.

The overall model for cognitive empathy and internalizing symptoms was also significant, *F*(4, 182) = 5.61, *p* <.001, predicting 17.65% of the variance. Results showed that parental mental illness moderated the effect between cognitive empathy and internalizing symptoms significantly, Δ*R*² = 1.78%. Whereas in COPMI cognitive empathy positively predicted internalizing symptoms, *b* = .249, 95% BCa CI [.032,.466], *t* = 2.723, *p* = .024, in COPWMI the relationship was not significant, *b* = -.092, 95% BCa CI [-.354,.171], *t* = -.688, *p* = .493. For cognitive empathy as independent variable and externalizing symptoms as outcome variable, the overall model was significant, *F*(4, 182) = 5.02, *p* <.001, predicting 9.94% of the variance. Results showed that parental mental illness moderated the effect between cognitive empathy and externalizing symptoms significantly, Δ*R*² = 3.23%. Whereas in COPMI cognitive empathy positively predicted externalizing symptoms, *b* = .299, 95% BCa CI [.090,.508], *t* = 2.826, *p* = .005, in COPWMI the relationship was not significant, *b* = -.125, 95% BCa CI [-.378,.129], *t* = -.971, *p* = .333. For regression coefficients, confidence intervals, standard errors, *p*-values, and test statistics see **Table 3** (affective empathy) and **Table 4** (cognitive empathy).

**Table 3:** Moderation analyses: Affective empathy and parental mental illness.

	Internalizing Symptoms					Externalizing Symptoms				
	*b*	95 CI	*SE*	*t*	*p*	*b*	95 CI	*SE*	*t*	*p*
Affective Empathy	.054	[-.222,.330]	.140	-.388	.264	-.025	[-.293,.244]	.136	-.182	.856
Parental Mental Illness	-.239	[-7.842, -7.364]	3.853	-.062	.951	-2.916	[-10.314, 4.482]	3.750	-.778	.438
Affective Empathy x Parental Mental Illness	.189	[-.210,.587]	.202	.934	.352	.259	[-.128,.647]	.197	1.320	.188
Covariate (SES)	-.214	[-1.020,.593]	.409	-.522	.602	-.015	[-.800,.771]	.398	-.037	.971
Constant	3.561	[-2.71, 9.830]	3.177	1.121	.264	4.243	[-1.858, 10.344]	3.092	1.372	.172

CI, confidence interval.

**Table 4:** Moderation analyses: Cognitive empathy and parental mental illness.

	Internalizing Symptoms					Externalizing Symptoms				
	*b*	95 CI	*SE*	*t*	*p*	*b*	*95 CI*	*SE*	*t*	*p*
Cognitive Empathy	-.092	[-.335, 11.729]	.133	-,688	.493	-.125	[-.378,.129]	.128	-.971	.333
Parental Mental Illness	-2.412	[-8.606, 3,782]	3.139	-.768	.443	-5.615	[-11.588,.357]	3.027	-1.855	.065
Cognitive Empathy x Parental Mental Illness	.341	[.001,.680]	.172	1.981	.049	.424	[.097,.751]	.166	2.556	.011
Covariate (SES)	-.203	[-1.006,.601]	.880	-.503	.616	-.025	[-.780,.749]	.393	-.064	.949
Constant	5.697	[-.335, 11.729]	3.057	1.863	.064	5.812	[-.004, 11.628]	2.948	1.972	.050

CI, confidence interval.

### 3.2 Empathy and psychopathology of children: the mediating effect of maladaptive ER strategies (aim 2)

**Figure 3:**
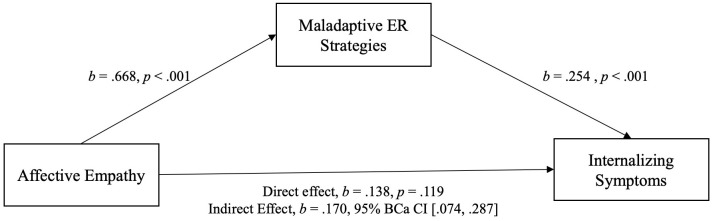
Model of affective empathy as a predictor of internalizing symptoms, mediated by maladaptive ER strategies. The confidence interval for the indirect effect is a BCa bootstrapped CI based on 5000 samples.

**Figure 4:**
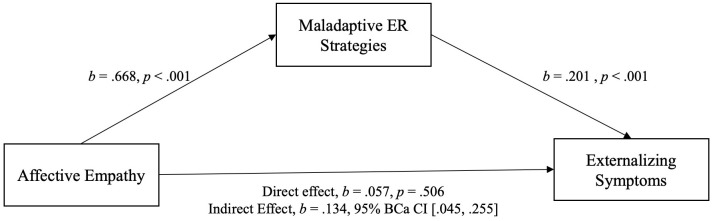
Model of affective empathy as a predictor of externalizing symptoms, mediated by maladaptive ER strategies. The confidence interval for the indirect effect is a BCa bootstrapped CI based on 5000 samples.

**Figure 5:**
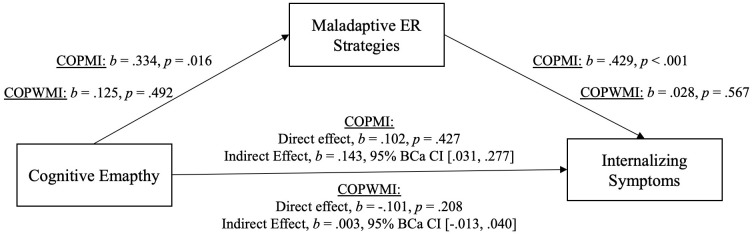
Model of cognitive empathy as a predictor of internalizing symptoms, mediated by maladaptive ER strategies. The confidence interval for the indirect effect is a BCa bootstrapped CI based on 5000 samples.

**Figure 6:**
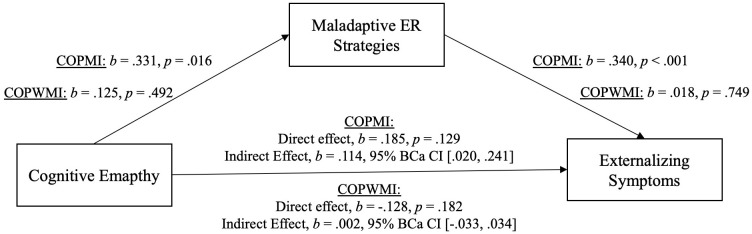
Model of cognitive empathy as a predictor of externalizing symptoms, mediated by maladaptive ER strategies. The confidence interval for the indirect effect is a BCa bootstrapped CI based on 5000 samples.

All variables of interest were correlated at the *p* < 0.01 level (see **Table 5**). Affective and cognitive empathy was positively moderately correlated with maladaptive ER and positively weak with internalizing/externalizing symptoms. Maladaptive ER was positively moderately associated with internalizing/externalizing symptoms. For demonstration of the results of the mediation analyses see **Figures 3**, **4** (affective empathy) and **Figures 5**, **6** (cognitive empathy).

**Table 5:** Correlation matrix of study variables, children (Total sample).

	Variables (children)	1	2	3	4	5	6	7
1	Cognitive Empathy	–						
2	Affective Empathy	.642**	–					
3	Maladaptive ER	.385**	.465**	–				
4	Internalizing symptoms	.273**	.313**	.441**	–			
5	Externalizing symptoms	.215**	.202**	.336**	.556**	–		
6	General psychopathology	.198**	.202**	.412**	.839**	.870**	–	
7	SES	-.218**	-.300**	-.307**	-.247**	-.133	-.179*	–

SES, Socioeconomic status.

*p <.05; **p <.01.

The relationship between affective empathy and psychopathological symptoms was not moderated by parental mental illness and psychopathological symptoms were significantly predicted by affective empathy in the total sample. We therefore assumed that affective empathy and psychopathological symptoms are related regardless of parental mental illness indicating the same relationship pattern in both groups. Consequently, we analyzed the mediating effect of maladaptive ER strategies in the total sample. The relationship between cognitive empathy and psychopathological symptoms was moderated by parental mental illness. Thus, we calculated two mediation analyses, one with COPMI and one with COPWMI.

Indeed, there was a significant indirect effect of affective empathy on psychopathological symptoms (inter-/externalizing symptoms) through maladaptive ER strategies. This indicates that the relationship between affective empathy and psychopathological symptoms can be explained by maladaptive ER in COPMI and COPWMI. Because of the significant moderating effect of parental mental illness on the relationship between cognitive empathy and psychopathological symptoms, two separate mediation analyses were calculated, one with COPMI and one with COPWMI each with internalizing and externalizing symptoms. In COPMI, there was a significant indirect effect of cognitive empathy on psychopathological symptoms (inter-/externalizing symptoms) through maladaptive ER strategies. This indicates that the relationship between cognitive empathy and psychopathological symptoms can be explained by maladaptive ER in COPMI. In COPWMI, neither the direct nor the indirect effect was significant indicating that cognitive empathy is unrelated to psychopathological symptoms in COPWMI.

## 4 Discussion

The purpose of the current study was to examine how empathy and psychopathology relate in COPMI vs. COPWMI and whether maladaptive ER mediate this relationship. We hypothesized that empathy (cognitive & affective) is positively related to psychopathological symptoms in COPMI and unrelated to psychopathological symptoms in COPWMI. In contrast to our expectation, the results differed for affective and cognitive empathy.

As expected, and in line with Tully and Donohue (24), increased cognitive empathy was only related to more internalizing symptoms in COPMI. In comparison to Tully and Donohue (24), we examined not only internalizing but also externalizing symptoms and found the same result for both, which indicate that in COPMI cognitive empathy is associated with inter- and externalizing symptoms. Just like in the study of Tully and Donohue (2019), in COPWMI cognitive empathy and psychopathology were not significantly related. This confirms the assumption, that parental mental illness can be a contextual factor contributing to maladaptive effects of cognitive empathy (24). Zahn-Waxler and van Hulle (33) have proposed that pathogenic guilt follows inaccurate assumptions of a causal role in the parent’s depression. Therefore, such guilt can contribute to internalizing symptoms and depression through empathy. Although Zahn-Waxler and van Hulle (33) made no distinction between affective and cognitive empathy. However, the proposed pathway suggests the relevance of rather cognitive than affective empathy since making assumptions about the own role in the parental mental illness includes cognitive processes. Indeed, Tully and Donohue (24) assigned this pathway to cognitive empathy. Since COPMI show as well more internalizing as externalizing symptoms (8, 9, 31), our results could indicate that the maladaptive pathway of cognitive empathy results in both groups of behavioral problems.

In contrast to cognitive empathy, parental mental illness did not moderate the relation between affective empathy and psychopathological symptoms. This result contradicts the theoretical literature insofar as Tully and Donohue (24) assumed both aspects of empathy to contribute to psychopathology in children of depressed mothers. Thus, it could be concluded that parental mental illness could be a moderator in both cases. However, studies with clinical and non-clinical samples have consistently shown that higher affective empathy is associated with higher internalizing symptoms (26, 27, 29, 73, 74). This indicates that heightened affective empathy is a risk factor for internalizing symptoms in general, i.e. independent of intraindividual moderators. Against the background of these studies, our results of the subsequently calculated linear regression analysis do not contradict the empirical research to date. The results namely indicates that affective empathy is associated with psychopathological symptoms. Thus, high affective empathy seems to be a risk factor regardless of the parental mental illness. Interestingly, we found the negative effect of affective empathy not only on internalizing but also externalizing symptoms. However, this is not surprising, since, as described below, the relationship between psychopathological symptoms (internalizing/externalizing symptoms) is explained by maladaptive ER strategies and these are also related to externalizing symptoms. In sum, the results indicate that especially in COPMI rather cognitive than affective empathy is a risk factor for psychopathological symptoms.

Secondly, we investigated whether the relation between empathy (affective & cognitive) and psychopathological symptoms is mediated by maladaptive ER strategies. Because parental mental illness did not moderate the association between affective empathy and psychopathological symptoms, we calculated this analysis with the total sample. In line with our hypothesis and MacDonald and Price (25), maladaptive ER strategies mediated the relationship. Since maladaptive ER is associated with both internalizing and externalizing symptoms (75), it makes sense that affective empathy leads indirectly not only to internalizing but also to externalizing symptoms. Further, the results suggest that not only young adults (25) but also children who are highly affectively empathic and are more likely to use maladaptive ER strategies in turn develop greater internalizing but also externalizing symptoms. Thus, the emotion state of an observer of another individual’s state is a function of the observer’s level of affective (and cognitive) empathy and is subject to the emotion regulatory process of the observer (48). The assumption is, that deficits in ER lead to higher levels of personal distress when confronted with another individual’s negative emotional state. Personal distress, in particular, is linked to a range of internalizing problems such as depression and anxiety (33, 76).

Our hypotheses regarding maladaptive ER strategies as mediator between cognitive empathy and psychopathological symptoms in COPMI was confirmed. There was neither a direct nor an indirect effect of cognitive empathy on psychopathological symptoms in COPWMI. The absence of the relation between cognitive empathy and psychopathological symptoms in COPWMI is in line with other studies with COPMI vs. COPWMI (24), inpatient adolescents (27), or young healthy adults (25). Previous studies investigating the relationship between cognitive empathy and difficulties in ER in adult community samples, showed a negative association suggesting that greater cognitive empathy can help to regulate negative emotions (25, 48). However, we examined this relationship in children at heightened risk for multiple psychological and developmental risks. Therefore, our results in COPMI are in line with theoretical literature suggesting that unfavorable conditions in the early family environment contribute to a maladaptive pathway of cognitive empathy (24, 33). Further, our results indicate that maladaptive ER may play a crucial role in the maladaptive pathway of cognitive empathy in COPMI. COPMI with high maladaptive ER strategies seem to try to explain their parents´ negative emotions but may attribute internally which can cause negative feelings such as guilt. The use of maladaptive ER strategies and ineffective regulation of negative feelings like guilt then result in psychopathological symptoms.

## 5 Strengths, limitations and implications

The main strength of this study is the differentiated analysis of associations between empathy, ER strategies and both internalizing and externalizing symptoms in children against the background of the contextual factor of parental mental illness. In this way, we integrated two relevant factors for the transgenerational transmission of psychopathology in one model and thus extended previous literature that is limited to single relationships or/and to investigations in adults (25). For instance, in adults, cognitive empathy has been negatively related to maladaptive ER (49, 77) and affective empathy was positively associated with maladaptive ER strategies (25). In turn, affective empathy was positively associated with internalizing symptoms in children (26, 29). However, no prior study has examined this pathway in one sample neither with COPMI nor COPWMI, such that mediation and indirect pathway between empathy and psychopathology could be pursued. Another strength is the clinical subsample in this study which enables stronger conclusions about the transgenerational effect of parental mental illness than studies based on community samples. Further, the present study is the first one to examine empathy and ER in COPMI of a clinical sample not limited to certain mental disorders like depression. Instead, our sample of parents had a wide range of psychopathology. This allows the generalization of the findings across mental disorders and the conclusion that cognitive empathy in particular seems to be a transdiagnostic mechanism of the TTMD through maladaptive ER. The large sample size and the representativeness of the clinical sample should also be positively emphasized. Beyond, the study is, along with one other (24), the only study to investigate parental mental illness as a moderator on the relation between empathy and psychopathology. It thus contributes to our understanding of the conditions under which empathy can be a “risky strength” (10).

Aside from these strengths, several limitations need to be mentioned. One limitation of the study is that parents reported the psychopathology for themselves and their children. Parent-ratings alone have been shown to be less valid for children’s internalizing symptoms but more valid for externalizing symptoms, at least in older age groups (70, 78). With regard to the psychopathological symptoms, it should be noted that the mean T-Scores of internalizing and externalizing symptoms and general psychopathology of both COPMI and COPWMI were in a normal range. In comparison to Loechner et al. (8) our COPMI sample had lower internalizing symptoms and general psychopathology but comparable externalizing scores. Wiegand-Greve et al. (9) report higher mean values on all main CBCL scales of COPMI than we, but also in a normal range. Another limitation is that both empathy and ER was assessed using self-report questionnaires. Since many processes associated with empathy seem to occur on an implicit level (47), authors raise concerns with using self-reported empathy as a valid predictor for actual performance (79). Regarding ER, previous literature recommended that ER should be studied as a multicomponent process including multiple types of measurement (e.g. self-report, behavior coding measure) (80). Moreover, in future studies objective measures, like psychophysiological measures, should be included to possibly solve the problem of inconsistent measures of empathy and ER across studies. Emotion regulation and empathy represent two promising mechanisms of TTMD. However, there are a number of other potential mediators and moderators that could play an important role in the relationships examined. Genetic transfer and parent-child interaction, for example, can be named as such here. A final limitation of the study is that the data are cross-sectional rather than longitudinal and therefore do not allow causal interpretations to be drawn about empathy and ER as factors prospectively predicting the onset of a mental disorder in COPMI. Thus, psychopathological symptoms could also cause more emotions and higher affective empathy. In order to clarify the direction of the relationship, capture developmental risks and model resilience for mental illness, longitudinal studies are needed. We are currently collecting data of further measurement points on the participants of the COPMI group in this study. This would allow us to address these questions. If prospective longitudinal research will support the present findings, they may have important implications for developing prevention and intervention programs for COPMI and thus interrupt the TTMD.

Our findings suggest that high affective empathy in children is associated with psychopathological symptoms and that this association is explained by maladaptive ER strategies. Whereas high affective empathy seems to be a risk factor for psychopathological symptoms in children in general, high cognitive empathy seems only to be risky for COPMI. These results highlight important clinical implications. First, it indicates that COPMI should receive preventive training in ER since it can be assumed that maladaptive ER is the mediating factor in the relationship between cognitive empathy and psychopathological symptoms. Particularly, it may be important to reduce the use of maladaptive ER strategies in COPMI. Second, assuming that in COPMI high cognitive empathy leads to pathogenic guilt, which in turn contributes to psychopathological symptoms, it would be important to reduce the pathogenic guilt. Preventive interventions for COPMI should therefore possibly include psychoeducational elements helping COPMI to understand the emotions, fluctuations of emotions and behaviors of their mentally ill parents. In this way, COPMI could also learn, that they are not responsible for the mental illness of their parents. Consequently, the attribution style of COPMI could be subject in preventive interventions. However, future studies should examine this theoretical pathway by measuring additionally pathogenic guilt and attribution style in COPMI and investigating them as mediating factors. The investigation of mediating factors, such as pathogenic guilt or attribution style, is particularly important for preventive interventions, as these can be changed.

## Data availability statement

The datasets presented in this article are not readily available because restrictions by law. Requests to access the datasets should be directed to christina.schwenck@psychol.uni-giessen.de.

## Ethics statement

The studies involving humans were approved by Local ethics committee of the University of Giessen. The studies were conducted in accordance with the local legislation and institutional requirements. Written informed consent for participation in this study was provided by the participants’ legal guardians/next of kin.

## Author contributions

AL: Conceptualization, Data curation, Formal analysis, Investigation, Methodology, Project administration, Software, Visualization, Writing – original draft, Writing – review & editing, Validation. KH: Data curation, Investigation, Project administration, Writing – review & editing. RuS: Supervision, Writing – review & editing. SW: Investigation, Supervision, Writing – review & editing. HC: Supervision, Writing – review & editing. MK: Data curation, Formal analysis, Methodology, Supervision, Writing – review & editing. KO: Supervision, Writing – review & editing. CR: Supervision, Writing – review & editing. RiS: Supervision, Writing – review & editing. LW: Supervision, Writing – review & editing. A-LZ: Supervision, Writing – review & editing. CS: Conceptualization, Funding acquisition, Investigation, Methodology, Resources, Supervision, Validation, Writing – review & editing.

## The COMPARE-family research group

Study management: Stracke, Gilbert, Eitenmüller. Biometry and data management: Awounvo, Kirchner, Klose. Clinical study monitoring: Buntrock, Ebert. Recruiting center Bielefeld: Schlarb. Recruiting center Bochum: Margraf, Schneider, Friedrich, Teismann. Recruiting center Gießen: Stark, Metzger. Recruiting center Greifswald: Brakemeier, Wardenga, Hauck. Recruiting center Landau: Glombiewski, Schröder, Heider. Recruiting center Mainz: Jungmann, Witthöft. Recruiting center Marburg: Rief, Eitenmüller.
